# Core shell hybrids based on noble metal nanoparticles and conjugated polymers: synthesis and characterization

**DOI:** 10.1186/1556-276X-6-98

**Published:** 2011-01-21

**Authors:** Ilaria Fratoddi, Iole Venditti, Chiara Battocchio, Giovanni Polzonetti, Cesare Cametti, Maria Vittoria Russo

**Affiliations:** 1 Department of Chemistry, University of Rome "Sapienza", P.le A.Moro 5, 00185 Rome, Italy; 2 Department of Physics, Unità INSTM and CISDiC University Roma Tre, Via della Vasca Navale 85, 00146 Rome, Italy; 3 Department of Physics, University of Rome "Sapienza", P.le A.Moro 5, 00185 Rome, Italy

## Abstract

Noble metal nanoparticles of different sizes and shapes combined with conjugated functional polymers give rise to advanced core shell hybrids with interesting physical characteristics and potential applications in sensors or cancer therapy. In this paper, a versatile and facile synthesis of core shell systems based on noble metal nanoparticles (AuNPs, AgNPs, PtNPs), coated by copolymers belonging to the class of substituted polyacetylenes has been developed. The polymeric shells containing functionalities such as phenyl, ammonium, or thiol pending groups have been chosen in order to tune hydrophilic and hydrophobic properties and solubility of the target core shell hybrids. The Au, Ag, or Pt nanoparticles coated by poly(dimethylpropargylamonium chloride), or poly(phenylacetylene-co-allylmercaptan). The chemical structure of polymeric shell, size and size distribution and optical properties of hybrids have been assessed. The mean diameter of the metal core has been measured (about 10-30 nm) with polymeric shell of about 2 nm.

## Introduction

The field of nanoscience and nanotechnology has found a dramatic attention in recent years and applicative perspectives of nanomaterials are widely studied [1]. One of the main goals in nanoscience is the understanding of materials behaviour when the size becomes close to atomic dimensions. Increased attention has been recently paid to metallic nanoparticles and in particular to noble metal nanoparticles (Au, Ag, Pt) that can be used in several fields: biomedicine, diagnostics [2], drug delivery systems [3], sensors [4,5], catalysis [6] and optics [7,8]. Optical tuneable properties have been deeply investigated [9] and arise from collective oscillation of conduction electrons within the nanoparticles resulting in the so-called plasmon resonance [10,11].

AuNPs have emerged as a broad new research field in the domain of colloids not only for their optical properties [12,13], but also for high chemical stability, catalytic use and size-dependent properties [14,15]. Aggregation phenomena can be avoided by protecting agents such as thiols or aminic compounds. Different synthetic protocols have been developed for the preparation of small, monodisperse nanoparticles [16,17]. One phase methods, based on organic solvents such as methanol [18] or tetrahydrofuran [19] have also been successfully developed. Thiol-protected AuNPs usually show high stability lasting even for years; recently Pd(II) containing organometallic thiols have also been used for the stabilization of AuNPs [20,21]. A number of functional groups such as thiopronin [19], succinic acid [22], sulfonic acid [23] and ammonium ions [24,25] have shown to result in stable and readily water dispersible AuNPs.

Silver nanoparticles (AgNPs) have gained interest over the years because of appealing properties, such as catalytic and antibacterial activity [26,27] which open perspectives in medical applications [28]. There are many methods for the synthesis as well as the control of the shape of AgNPs [29]. Silver nanoparticles can be synthesized by means of several methods and chemical reduction is one of the most frequently applied methods for their preparation as colloidal dispersions in water or organic solvents [30,31]. The reduction of silver ions in aqueous solution generally yields colloidal silver with particle diameters of several nanometres [32]. The synthesis is often carried out in the presence of stabilizers in order to prevent unwanted agglomeration of the colloids. Among others, tertiary amines have been recently used to form Ag nanoparticles in organic medium [33]. Amine derivative complexes have been used to synthesize Au nanoparticles as well [34,35].

Platinum metal is used in industrial catalysts and can be found in the catalytic converters, and platinum nanoparticles (PtNPs) have been recently used as a novel hydrogen storage medium [36]. Colloidal PtNPs are synthesized in a fashion similar to that of AuNPs and AgNPs, by reduction of H_2_PtCl_6 _in the presence of a citrate capping agent. Colloidal platinum can be functionalized with nucleic acids and has been used as label for the amplified biorecognition of DNA hybridization, aptamer/protein recognition events and tyrosinase activity [37]. Colloidally prepared Pt nanoparticles capped with organic ligands appear to be suitable as supported catalysts, and CO adsorption experiments have clearly shown that small molecules can pass through the ligand shell and adsorb on free areas of the Pt surface [38].

There has been recently a strong interest in the self-assembly of metal nanoparticles into ordered structures, mainly by using bifunctional molecules such as organic dithiols [39], surfactants [40] and polymers [41]. Noble metal nanoparticles protected by synthetic polymers, i.e. core shell systems, are envisioned to be superior to polymeric micelles, for example as thermosensitive materials for biomedical applications [42]. Metal nanoparticles stabilized by polymers can be prepared by postmodification of preformed gold nanoparticles and physisorption [43] or by "graft-from" and "graft-to" methods. For example, surface-initiated atom transfer radical polymerization technique has been successfully used to modify Ni nanoparticles and poly(methylmethacrylate) and poly(*n*-isopropylacrylamide) were grafted from the immobilized initiators [37]. A facile approach to prepare thiol-terminated poly(styrene-ran-vinyl phenol) (PSVPh) copolymers and PSVPh-coated gold nanoparticles is reported with the goal of creating stabilizing ligands for nanoparticles with controlled hydrophilicity [44]. Polymer shells have been formed around AgNPs by polymerization of adsorbed and solution-free monomers [45,46] and the reduction of Ag salts in polymer micelles [47]. Both hydrophilic and hydrophobic polymers [48,49] have been tested and the development of synthesis protocols has received considerably attention. Water-dispersible metal nanoparticles are expected to have applications in catalysis, sensors, molecular markers and in particular, biological applications such as biolabelling and drug delivery.

In this paper, the synthesis and characterization of core shell systems based on noble metal nanoparticles and hydrophilic and hydrophobic polymer shells are reported. In particular, the "graft-to" strategy was applied starting from the ammonium-containing conjugated polymer, i.e. poly(dimethylpropargylamonium chloride) [P(DMPAHCl)] and a thiol-containing co-polymer, poly(phenylacetylene-co-allylmercaptan) [P(PA-co-AM)]. The polymers were used as stabilizer during the generation of Au, Ag and Pt nanoparticles and the materials were fully characterized by means of basic spectroscopic techniques, dynamic light scattering (DLS), *Z*-potential and X-ray photoelectron spectroscopy (XPS) and, for the investigation of morphology and dimensions of self-assembled structures, by transmission electron microscopy (TEM) techniques.

## Experimental

### Materials

Gold(III) chloride trihydrate (HAuCl_4 _3H_2_O) (99.9%), silver nitrate (AgNO_3_) (99.9%), potassiumtetrachloroplatinate(II) (K_2_PtCl_4_) (99.9%), tetra-*n*-octylammonium bromide (TOAB) (98%), sodium borohydride (NaBH_4_) (98%), 3-dimethylamino-1-propyne (DMPA) (98%), phenylacetylene (PA) (98%), allylmercaptane (AM) (98%), potassium persulphate (99%), toluene, ethanol, and chloroform were purchased from Sigma Aldrich. All reagents were used as received without further purification. Water was purified through a Millipore-SIMPAKOR1system (Simplicity 185) and degassed for 30 min with Argon, before use. Conjugated polymer P(DMPAHCl) was synthesized in analogy to the method reported in our previous work [50], using Rh(I) dimer complex [Rh(cod)Cl]_2 _(cod = cyclooctadiene) with complex/monomer ratio 1/100 (a typical procedure is reported in Additional file 1). P(PA-co-AM) was prepared by using the emulsion polymerization technique in analogy to the synthesis of similar copolymers reported in our recent paper [51], with co-monomer ratios PA/AM = 5/1 and 10/1 (a typical procedure is reported in Additional file 1, together with the main characterizations of the precursor polymers).

### Synthesis of hydrophilic metal nanoparticles

The hydrophilic metal core shell systems (Au, Ag, Pt) were prepared using the following procedure: gold(III) chloride trihydrate (0.02 g, 0.051 mmol) or silver nitrate (0.02 g 0.118 mmol) or potassiumtetrachloroplatinate (0.02 g 0.048 mmol) was dissolved in water (10 ml) to form a clear solution to which the polymer solution was then added (0.02 g of P(DMPAHCl) in 10 ml water). The mixture was vigorously stirred and degassed with Ar for 15 min. A water solution of sodium borohydride (0.02 g in 10 ml) was put into the mixture slowly. The reaction was stopped after 12 h and the water phase was left overnight in freezer (-20°C); the next day the dark precipitate, i.e. Au@P(DMPAHCl), Ag@P(DMPAHCl) or Pt@P(DMPAHCl), was washed several times with water by centrifugation and finally dried at 40°C (Yield 35 wt%). Main characterizations: Au@P(DMPAHCl): IR (film, cm^-1^):1615, 1250, 1120; UV-Vis (CHCl_3_): λ_max _= 296, 540 nm; Ag@P(DMPAHCl): IR (film, cm^-1^):1615, 1250, 1120; UV-Vis (CHCl_3_): λ_max _= 296, 410 nm; Pt@P(DMPAHCl): IR (film, cm^-1^):1615, 1250, 1120; UV-Vis (CHCl_3_): λ_max _= 300 nm.

### Synthesis of hydrophobic metal nanoparticles

The hydrophobic metal (Au, Ag) nanoparticles were prepared by the following route: gold(III) chloride trihydrate (0.02 g, 0.05078 mmol) or silver nitrate (0.02 g, 0.1177 mmol) was dissolved in water (20 ml) to form a clear yellow solution, then polymeric solution (0.01 g P(PA-co-AM) in 10 ml toluene) and TOAB in toluene solution (0.035 mg in 4 ml) were added. The mixture was vigorously stirred and degassed with Ar for 15 min at room temperature. A water solution of sodium borohydride (0.02 g in 10 ml) was added to the mixture drop-by-drop. The reaction was allowed to react and maintained under stirring for 12 h. The black product, i.e. Au@P(PA-co-AM) or Ag@P(PA-co-AM) was extracted with a separator funnel two times with water (10 ml each) and, after that, the organic phase was left overnight in freezer (-20°C); the next day the dark precipitate was washed several times by centrifugation with ethanol and finally dried at 40°C (Yield 25 wt%). Main characterizations: Au@P(PA-co-AM): IR (film, cm^-1^): 3050, 2580, 1597; UV-Vis (CHCl_3_): λ_max _= 525 nm; Ag@P(PA-co-AM): IR (film, cm^-1^): 3050, 2580, 1597; UV-Vis (CHCl_3_): λ_max _= 400 nm.

### Instruments

UV-Vis spectra were recorded on a VARIAN Cary 100. All optical measurements were performed at room temperature using quantitative H_2_O or CHCl_3 _solutions. NMR spectra were recorded on a Varian XL-300 spectrometer at 300 MHz, in appropriate solvents (CDCl_3_, D_2_O); the chemical shifts (ppm) were referenced to TMS for ^1^H NMR assigning the residual ^1^H impurity signal in the solvent at 7.24 ppm (CDCl_3_). Molecular weights were determined at 25°C by gel permeation chromatography on a PL-gel column containing a highly cross-linked polystyrene/divinylbenzene matrix packed with 10 μm particles of 100 Å pore size using CHCl_3 _(HPLC grade) as eluent (details in Additional file 1). Samples for TEM measurement were prepared by placing a drop of suspension onto a carbon-coated copper grid and examined using a Philips CM120 Analytical transmission electron microscope with LaB6 filament, operating at 120 kV, magnification up to 660.000 ×, resolution up to 0.2 nm. DLS measurements were carried out using a Brookhaven instrument (Brookhaven, NY, USA) equipped with a 10 mW HeNe laser at a 632.8 nm wavelength, at the temperature of 25.0 ± 0.2°C. Correlation data were collected at 90° relative to incident beam and delay times from 0.8 μs to 10 s were explored. Correlation data were fitted using the non-negative least squares or CONTIN algorithms [52,53], supplied with the instrument software. The average hydrodynamic radius *R*_H _of the diffusing objects was calculated from the diffusion coefficient *D *and the Stokes-Einstein relationship, *R*_H _= (*K*_B_*T*)/(6πη*D*), where *K*_B_*T *is the thermal energy and η is the solvent viscosity. XPS spectra were obtained using a custom-designed spectrometer. A non-monochromatic MgKα X-rays source (1253.6 eV) was used and the pressure in the instrument was maintained at 1 × 10^-9 ^Torr throughout the analysis; binding energies (BE) were corrected by adjusting the position of the C1s peak to 285.0 eV in those samples containing mainly aliphatic carbons and to 284.7 eV in those containing more aromatic carbon atoms, in agreement with literature data [54] (see details in Additional file 1).

## Results and discussion

**Figure 1:**
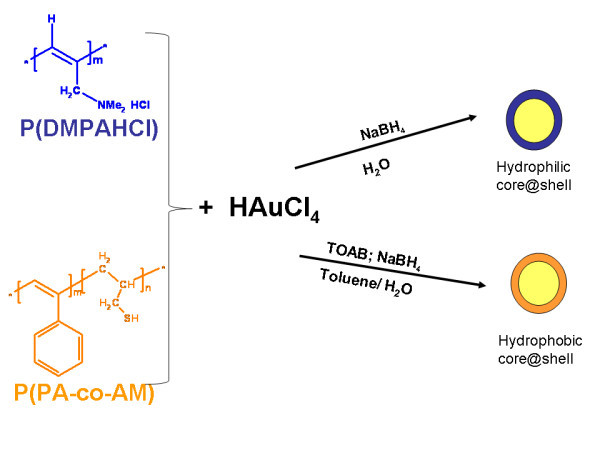
**Typical procedure to obtain hydrophobic and hydrophilic core shell hybrids**.

The preparation of hydrophilic and hydrophobic core shell hybrids has been carried out by performing wet reductions of metal salts in the presence of polymeric solutions (see Figure 1: The chemical synthesis of Au core shell hybrids, reported as an example).

The size and shape of the nanoparticles prepared by the reduction of the ions in solution normally depends on a number of parameters, such as the kind of reducing agent and the loading of the metal precursor. The reducing agent determines the rate of nucleation and particle growth: slow reduction produces large particles, while fast reduction gives small particles. In every case the NaBH_4 _was chosen as the reducing agent, which leads to a fast rate of nucleation and usually small metal cores.

In the case of P(DMPAHCl)-based core shell systems, due to their high water solubility, the reaction was carried out in aqueous phase, without the need of TOAB stabilizer. On the other hand, in the case of hydrophobic P(PA/AM)-based systems, a classical two phase procedure has been used allowing the TOAB to act as the phase transfer from the organic to the aqueous one.

**Figure 2:**
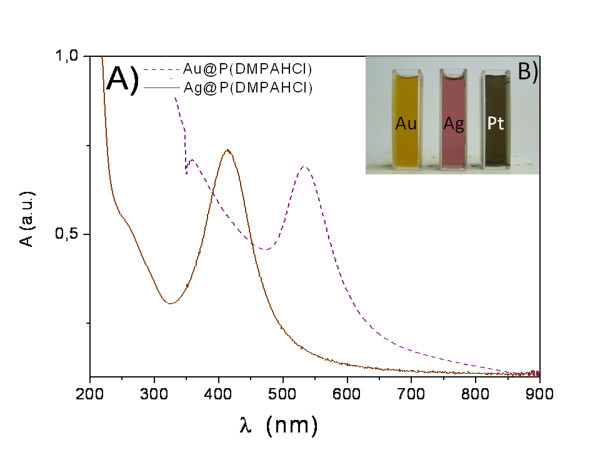
**a: UV-Vis absorption spectra of samples Au@P(DMPAHCl) and Ag@P(DMPAHCl) and b: Ag, Au and Pt core shell in water suspensions image (yellow, pink and grey, respectively)**.

Metal core shells have been produced from the reduction of AuCl^-^, Ag^+ ^or PtCl_4_^2- ^ions in aqueous solutions in the presence of polymers. In the case of gold, upon addition of NaBH_4 _the colour of the solution gradually turned from yellowish to clear to grey to purple during the reaction, indicating the formation of small gold nanoparticles. The progress of the reaction leading to Au and Ag-based core shell hybrids has been monitored following their plasmon absorption bands, whereas the Pt nanoparticles evolution have been recorded from the growth of the featureless absorption bands, monotonically increasing in the visible region. Representative UV-Vis spectra of the samples Au@P(DMPAHCl), and Ag@P(DMPAHCl) are shown in Figure 2, together with an image of the water suspensions.

The characteristic plasmon band for gold and silver has been observed at about 540 and 410 nm, respectively, with shoulders at about 300 nm, due to the absorption of polymeric shell. As expected, in the spectra of PtNPs, recorded at the end of the reaction, no characteristic peaks of the nanoparticles have been observed and a broad absorption at about 300 nm has been assigned to the polymer shell. During the evolution of the metal nanoparticles, UV-Vis spectra of the metal sols at different times have also been recorded and it was found that as the time progresses the absorption bands for Au and Ag narrowed and shifted continuously to the shorter wavelength regions. Purification of the nanoparticles has been performed by centrifugation of the pristine suspension, giving rise to samples a, b, c with the characteristic plasmon band split in two components, centred at 540 and 695 nm (sample Au@P(DMPAHCl-c). This behaviour can be explained as a consequence of the isolation of core shell hybrids with different shapes, sizes, and compositions. While gold nanospheres usually show one absorption band in the visible region, gold nanorods are reported to show two bands [55]. The IR spectra of the core shell hybrids show the characteristic features of the structural units of the polymeric shell, not affected by the reduction procedures, thus confirming the achievements of a defined and stable polymeric shell.

In the case of Au@P(PA-co-AM) and Ag@P(PA-co-AM) samples, upon addition of NaBH_4 _to AuCl_4_^- ^solution in the presence of P(PA/AM) copolymer, the colour of the solution rapidly turned to brown during the reaction and UV-Vis spectra of the purified samples show the characteristic plasmon band of gold and silver at about 525 and 400 nm, partially overlapped the typical large absorption band of the P(PA-co-AM) copolymer at about 370 nm. Also in this case the IR characterization confirmed the presence of the functional group characteristics of the polymeric shell.

XPS characterization has been carried out on our materials and allowed to investigate the interaction at the interface between metal nanoclusters and polymers, as well as the chemical composition of the resulting core shell materials. C1s, N1s, S2p, Cl2p and Au4f, Ag3d or Pt4f signals have been acquired. For comparison, pristine P(DMPAHCl) and P(PA-co-AM) polymers were also investigated.

C1s spectra of all samples appear structured and three components were individuated by peak fitting: a main signal at 285.0 eV due to aliphatic carbon atoms that was used for the calibration procedure (see "Experimental" section), a component at about 286.5 eV belonging to C atoms bridged to aminic (C*-N) or thiol (C*-S) groups, and a third signal of very low intensity at higher BE values (288.5 eV) that is due to organic contaminants chemisorbed on the sample surface. Metal XPS spectra, i.e. Au4f, Ag3d and Pt4f, show a couple of spin orbit pairs. The signal at lower BE values (83.80 eV for Au4f7/2, 369.07 eV for Ag3d5/2 and 73.49 eV for Pt4f7/2) was assigned to metallic gold, silver and platinum, respectively; the feature at higher BE values (84.65 eV for Au4f7/2, 369.80 eV for Ag3d5/2 and 74.91 eV for Pt4f7/2) was attributed to metal atoms interacting with the co-polymer functional group, i.e. -N(CH_3_)_2 _for P(DMPAHCl) and -SH for P(PA-co-AM). The direction of the shift in metal XPS spectra clearly indicates that part of the metal atoms are in an oxidized state, i.e. the metal-polymer interaction causes a decreased electron density on the interacting metal atoms. For example, a BE value of 84.6 eV for Au4f7/2 component is consistent with the BE value of 84.4 eV reported in the literature for Au(1) complexes [56]. N1s spectra of Au@P(DMPAHCl), Ag@P(DMPAHCl) and Pt@P(DMPAHCl) revealed two components at about 400.2 and 402.5 eV. The signal at higher BE values was attributed to the unperturbed aminic groups, by comparison with the pristine P(DMPAHCl) polymer. The N1s spectrum of P(DMPAHCl) shows a single signal at about 402.3 eV, as expected for aminic groups interacting with Cl^- ^ions, alike for example in NH_4_X or (CH_3_)_4_NX [57]; Cl2p spectra were also collected and the observed Cl2p3/2 signal is found at about 197.80 eV in both pristine polymers and core shell systems, and attributed to Cl^- ^ions alike for NH_4_Cl [58]. The second N1s peak observed for the core shell M@P(DMPAHCl) samples at 400.2 eV was assigned to aminic groups bonded to Au and, respectively, Ag and Pt. The observed decrease in N1s BE value is related to the increased charge density on N atoms, as a consequence of the nitrogen-metal interaction. A completely similar behaviour was observed for S-containing polymers grafting Au and Ag nanoparticles in Au@P(PA-co-AM) and Ag@P(PA-co-AM), where S2p3/2 signal BE decreases from 163.2 to about 162.0 eV going from pristine P(PA-co-AM) co-polymer to the core shell systems. A completely similar trend was observed for thiols anchored on metal nanoclusters as well as metal surfaces, and extensively discussed in the literature [59,60]. The above discussed XPS analysis lead to ascertain that a covalent bond occurs between the metal atoms and the polymer functional group, DMPA (N atoms) and AM (S atoms), respectively.

**Figure 3:**
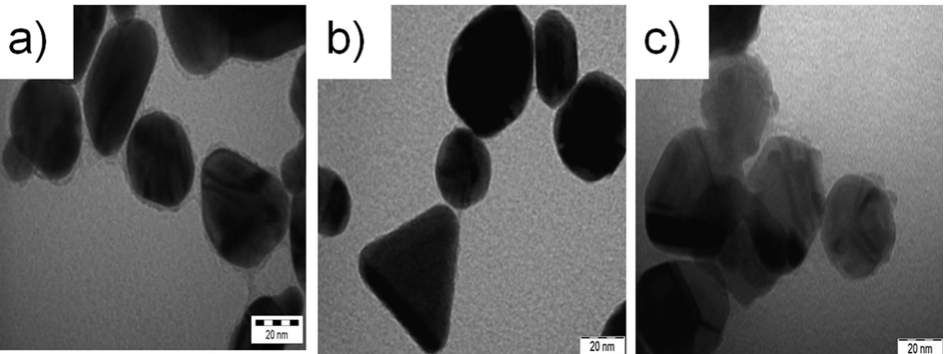
**TEM image of core shell hybrids**: **(a) **Au@P(DMPAHCl); **(b) **Au@P(DMPAHCl)-c; **(c) **Ag@P(DMPAHCl).

**Figure 4:**
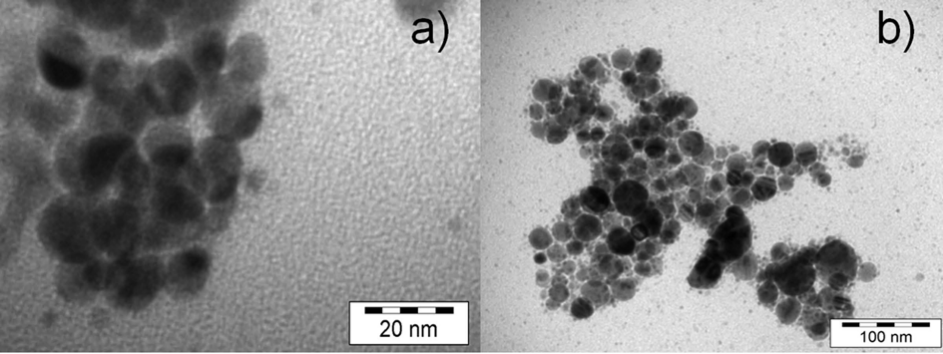
**TEM image of**: **(a) **Au@ P(PA-co-AM); **(b) **Ag@P(PA-co-AM).

Inspection of the TEM image revealed different shapes of the core shell structure of the polymer-stabilized metal nanoparticles. In the case of Au@P(DMPAHCl) (shown in Figure 3a), the average diameter of the gold cores was less than 30 nm, surrounded by a polymer shell with a thickness of about 2 nm. A selected sample, i.e. Au@P(DMPAHCl)-c was also studied and revealed the presence of different shapes ranging from triangles to rods with dimensions in the range 20-40 nm. A detail of the structure is shown in Figure 4b. Ag-based nanoparticles showed generally an hexagonal shape with mean dimension of about 30 nm (Figure 4c), whereas smaller diameters have been observed for Pt-based nanoparticles (less than 20 nm), that appear to be formed of smaller particles with irregular shapes.

In Figure 4a,b the TEM images of Au@P(PA-co-AM) and Ag@ P(PA-co-AM) obtained from P(PA-co-AM) with co-monomer ratio 5/1, are reported. In this case dispersed nanoparticles have been observed and the dimensions are distributed in the range of 5-15 nm for AuNPs and 10-30 nm for AgNPs.

**Figure 5:**
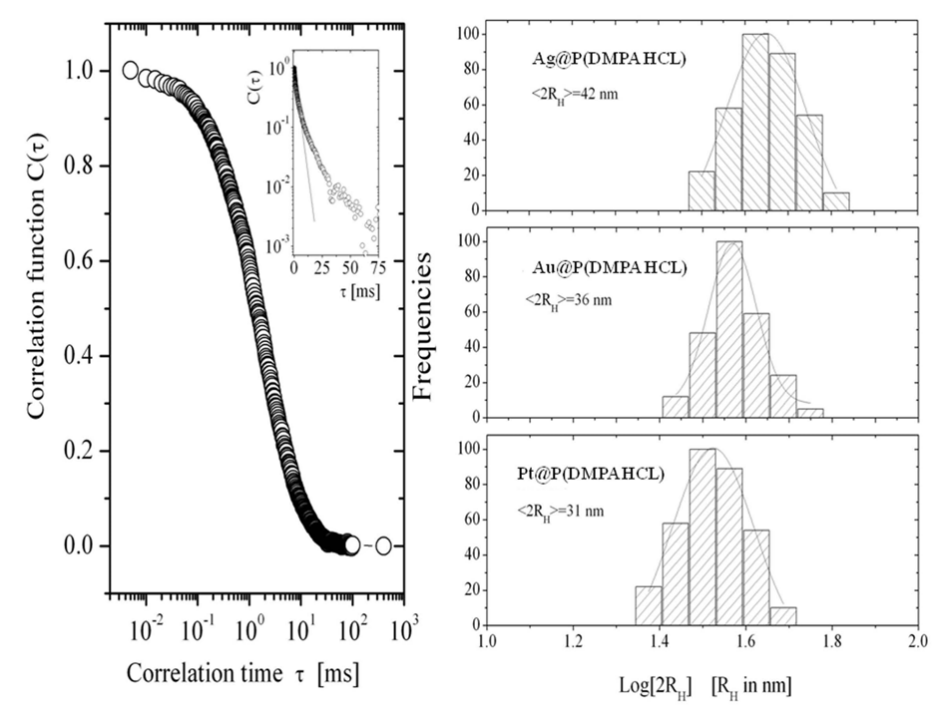
**DLS measurements**.**Left**: A typical correlation function C(τ) as a function of the correlation time τ for Ag(DMPAHCl) in aqueous solution. The inset shows the correlation function in a log scale, evidencing deviations, at longer times, from a single relaxation process characterized by a single decay time. **Right**: The histograms of the distribution of the hydrodynamic radius of the nanoparticles in aqueous solutions. **Upper panel**: Ag@P(DMPAHCl) with an average hydrodynamic radius of 21 ± 2 nm; **intermediate panel**: Au@P(DMPAHCl), with an average hydrodynamic radius of 18 ± 2 nm; **bottom panel**: Pt@P(DMPAHCl), with an average hydrodynamic radius of 15.5 ± 0.5 nm.

The size and size distribution of hydrophobic core shell hybrids in aqueous solutions have been investigated by means of DLS measurements resulting in a hydrodynamic radius around 15-20 nm with a relatively low dispersity as determined by the first cumulant analysis. We obtained an average hydrodynamic radius of 21 ± 2 nm for Ag@P(DMPAHCl); a value of 18 ± 2 nm for Au@P(DMPAHCl), and a value of 15.5 ± 0.5 nm for Pt@P(DMPAHCl). For all the samples investigated, the dispersity varies in the range of 0.08-0.15 (see Figure 5). These values are in a fairly good agreement with TEM measurements.

**Figure 6:**
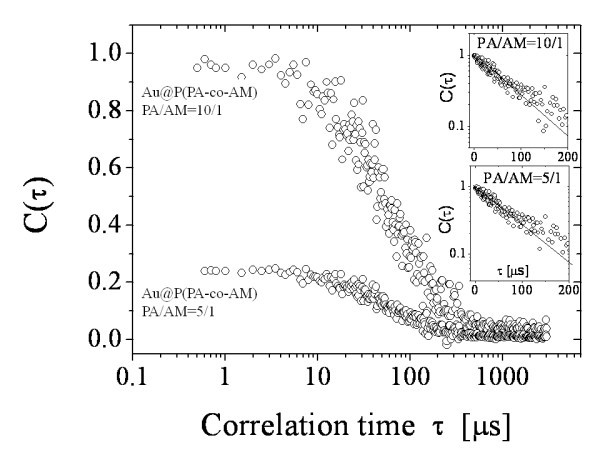
**The autocorrelation functions of Au-NPs in CHCl_3 _solutions at two different PA/AM ratios of copolymeric shell, as a function of the correlation time**. The insets show the analysis of the autocorrelation functions by means of the cumulant method to emphasize deviation from a single exponential decay due to the dispersity.

A similar behaviour was observed in the case of hydrophobic, i.e. with P(PA-co-AM) shell in CHCl_3 _solution. A typical example of the correlation functions for Au@P(PA-co-AM) is shown in Figure 6 with two different ratios of P(PA-co-AM) polymeric shell. In these conditions, the average hydrodynamic radius is 20 ± 2 nm for PA/AM = 10/1 and 22 ± 3 nm for PA/AM = 5/1.

The reported results show the achievement of an easy and versatile synthesis of core shell systems based on noble metal nanoparticles that allows the modulation of morphology, dimensions and chemical-physical properties of these nanoparticles, such as the hydrophilic-hydrophobic character, using an appropriate conjugated polymeric shell.

## Conclusions

A versatile and facile synthesis of core shell systems based on noble metal nanoparticles (AuNPs, AgNPs, PtNPs), coated by polymers and copolymers belonging to the class of substituted polyacetylenes has been developed. The polymeric shells containing different functionalities have been chosen in order to tune the hydrophilic and hydrophobic properties of the target core shell hybrids. The core shell dimensions can be tailored by the synthesis and obtained in the range of 10-30 nm. The nanoparticles show hydrophilic and hydrophobic groups on the surface of the spherical shell and this functional property is a suitable tool for future applications of these coated metal nanoparticles for biomedicine and sensors.

## Abbreviations

AgNPs: silver nanoparticles; AM: allylmercaptane; BE: binding energies; DLS: dynamic light scattering; PA: phenylacetylene; P(DMPAHCl): poly(dimethylpropargylamonium chloride); P(PA-co-AM): poly(phenylacetylene-co-allylmercaptan); PSVPh: poly(styrene-ran-vinyl phenol); PtNPs: platinum nanoparticles; TEM: transmission electron microscopy; XPS: X-ray photoelectron spectroscopy.

## Competing interests

The authors declare that they have no competing interests.

## Authors' contributions

IV, IF and MVR carried out the synthesis and characterizations and drafted the manuscript, CC light scattering characterizations, CB and GP carried out XPS studies. All authors read and approved the final manuscript.

## Supplementary Material

Additional file 1 **Supporting information**. A Word DOC containing supporting information. Click here for file
