# Supplementary material for: Core shell hybrids based on noble metal nanoparticles and conjugated polymers: synthesis and characterization
Source: Nanoscale Res Lett. 2011 Jan 21;6(1):98. doi: 10.1186/1556-276X-6-98 (PMC3212248; doi:10.1186/1556-276X-6-98)
Supplement: Additional file 1 — Supporting information. A Word DOC containing supporting information. [file 1556-276X-6-98-S1.DOC]

**SUPPORTING INFORMATION**

***Synthesis of poly(dimethylpropargylamonium chloride), P(DMPAHCl)***

Polymer PDMPAHCl was obtained by reaction of 2 g (0.0167 mol) of monomer DMPAHCl in the presence of 0.00167 mol of [Rh(cod)Cl]_2_ complex, in 20 mL of buthanol as the solvent. The mixture was stirred at 120 °C for 24h and the reaction solvent was eliminated under vacuum. Hexane was added to the crude product to obtain a brown powder that was filtered and washed with n-hexane and THF.

***Synthesis of poly(phenylacetylene-co-allylmercaptan), P(PA-co-AM)***

Deionized water (50 mL), toluene (1 mL), PA (1 mL, 9.16 mmol), and AM (0.2 mL, 2.5 mmol) were mixed and degassed for 15 min under stirring in Argon atmosphere, and then the temperature raided at 80 °C for 1 h. A water solution of KPS (50 mg in 5 mL) was added and the reaction was refluxed under stirring in Ar atmosphere for 20 h. The polymerization was stopped by opening the flask and the light yellow emulsion was filtered, centrifuged at 2000 rpm and re-dispersed in deionized water several times, in order to remove all un-reacted chemicals and toluene (weight yield 60%).

***Main characterizations of the starting polymers:***

P(DMPAHCl): Total yield: 89 %. IR (film, cm^-1^):1620, 1250, 1120; UV-vis (CHCl_3_): λ_max_ = 300 nm; ^1^H-NMR (CDCl_3_ ppm): 1.80 (m, NCH_3_), 2.88 (m, CH_2_N), 5.30 (m, =CH _chain_), 11.78 (m, N-H). M_w_ = 59000; p= 1.8

P(PA/AM); IR (film, cm^-1^): 2580; 1622; 760. UV-vis (CHCl_3_): λ_max_ = 370 nm; ^1^H NMR (300 MHz, CDCl_3_, δ): 0.81(m, CH_2_,_chain_), 1.19 (m, CH_2_,_chain_), 1.51(m, SH), 2.04 (m, CH_2_S), 5.20 (m, =CH _chain_), 7.4-7.3 (dd, Ph); GPC: M_w_ =1100, p= 1.2

Instruments details

Permeation Chromatography (GPC) on a PL-gel column containing a highly cross-linked polystyrene/divinylbenzene matrix packed with 10 micron particles of 100 Å pore size using CHCl_3_ (HPLC grade) as eluent and pumped at a flow-rate of 0.8 ml/min by a binary LC pump, via an injection loop of volume 24 μL. Monodisperse polystyrene standards were used for calibration, and the samples detected by UV at 300 nm.

X-ray photoelectron spectroscopy (XPS) spectra were obtained using a custom designed spectrometer. The experimental apparatus consists of an analysis chamber and a preparation chamber separated by a gate valve, resolving power 0.5 x10^-3^. The instrument is equipped with an electrostatic hemispherical analyser (mean radius 150 mm) operating in the fixed analyser transmission (FAT) mode and a 16-channel detector was used giving a total instrumental resolution of 1.0 eV as measured at the Ag (3d_5/2_) core level. The film samples were prepared by dissolving our materials in CHCl_3_ and spinning the solutions onto polished stainless steel substrates. The samples were stable during the XPS analysis, preserving the same spectral features and chemical composition. Curve-fitting analysis of the C1s, S2p, Au4f, Ag3d, Pt4f, and Cl2p experimental signal was performed using Voigt profiles as fitting functions, after subtraction of a Shirley-type background [D.A. Shirley, Phys. Rev. B 1972, 5, 4709-4714] using the Peak Fit curve fitting program for PC. Quantitative evaluation of the atomic ratios was obtained by analysis of the XPS signal intensity, employing Scofield’s atomic cross section σ values [J.M. Scofield, J. Electron Spectrosc. Relat. Phenom. 1976, 8, 129-137] and experimentally determined sensitivity factors.
